# The prevalence, metabolic risk and effects of lifestyle intervention for metabolically healthy obesity: a systematic review and meta-analysis: A PRISMA-compliant article

**DOI:** 10.1097/MD.0000000000008838

**Published:** 2017-11-27

**Authors:** Hanli Lin, Liqun Zhang, Ruizhi Zheng, Yishan Zheng

**Affiliations:** a Department of Cardiology; b Department of Intensive Care Unit, Zhejiang Putuo Hospital, Zhoushan; c Department of Epidemiology and Statistic, Zhejiang University, Hangzhou, Zhejiang; d Department of Intensive Care Unit, The Second Hospital of Nanjing. Teaching Hospital of Medical School of Nanjing University, Nanjing, Jiangsu, China.

**Keywords:** intervention, meta-analysis, metabolic, obesity, prevalence

## Abstract

**Background:**

We conducted a systematic review and meta-analysis to firstly obtain a reliable estimation of the prevalence of metabolically healthy obese (MHO) individuals in obesity, then assessed the risk of developing metabolic abnormalities (MA) among MHO individuals. At last, we evaluated the effects of traditional lifestyle interventions on metabolic level for MHO subjects.

**Methods:**

A systematic review and meta-analysis (PRISMA) guideline were conducted, and original studies were searched up to December 31, 2016. The prevalence of MHO in obesity from each study was pooled using random effects models. The relative risks (RRs) were pooled to determine the risk of developing MA for MHO compared with metabolically healthy normal-weight (MHNW) subjects. For the meta-analysis of intervention studies, the mean difference and standardized mean differences were both estimated for each metabolic parameter within each study, and then pooled using a random-effects model.

**Results:**

Overall, 40 population-based studies reported the prevalence of MHO in obesity, 12 cohort studies and 7 intervention studies were included in the meta-analysis. About 35.0% obese individuals were metabolically healthy in the obese subjects. There were dramatic differences in the prevalence among different areas. However, 0.49 (95% confidence intervals [CI]: 0.38 to 0.60) of the MHO individuals would develop one or more MA within 10 years. Compared with MHNW subjects, the MHO subjects presented higher risk of incident MA (pooled RR = 1.80, 95%CI: 1.53–2.11). Following intervention, there was certain and significant improvement of metabolic state for metabolically abnormal obesity (MAO) subjects. Only diastolic blood pressure had reduced for MHO individuals after intervention.

**Conclusions:**

Almost one-third of the obese individuals are in metabolic health. However, they are still at higher risk of advancing to unhealthy state. Therefore, it is still needed to advise MHO individuals to maintain or adopt a healthy lifestyle, so as to counterbalance the adverse effects of obesity.

## Abstract 2

Supplemental Digital Content is available in the text

## 1 Introduction

Obesity now represents one of the major health problems in the world for its vital contribution to diabetes and cardiovascular disease.^[1]^ However, it has been suggested that diseases risk associated with obesity may not be uniform, a subgroup of obese individuals do not present metabolic syndrome, referred as metabolically healthy obesity (MHO).^[2]^ In different investigations, the prevalence of MHO varied, which was influenced by gender, age, race and the definition and metabolic health.^[2]^ Longitudinal studies have confirmed that, compared with metabolically healthy normal-weight (MHNW) subjects, MHO individuals are still at increased risk for diabetes and cardiovascular diseases; however, the risks are much lower than that of metabolically abnormal obese (MAO) counterparts.^[3,4]^ On the other hand, it has been reported that MHO is not a permanent state and many of these individuals will convert to metabolically unhealthy status, with reluctant risk of major diseases.^[5,6]^ Therefore, it is generally considered that MHO is a temporally intermediate stage on the pathway to developing metabolic syndrome.

To reduce the risk of developing major diseases for MHO individuals, probably it is needed to take necessary measures to stop the deterioration in metabolic function for them. It had been reported that obese adults respond differently to dietary intervention or physical activity intervention for weight loss.^[7]^ Therefore, it is not completely clear if MHO individuals would benefit from traditional lifestyle interventions, and previous studies about lifestyle intervention in MHO patients had obtained conflicting results. Two intervention studies showed an improvement in cardiometabolic profile in MAO, but not in MHO individuals, despite both of them presenting similar weight loss.^[8,9]^ Nevertheless, the intervention study conducted by Ruiz et al^[10]^ suggested that MHO women also benefit from a 12-week energy-restricted diet intervention.

Rey-López et al^[11]^ had summarized that the percentage of metabolic health individuals in obesity ranged between 6% and 75%. Wang et al^[12]^ reported the prevalence of MHO in general population was 7.27%. After that, several studies also published their results with large sample size,^[13–15]^ especially for the investigations conducted in Cameroon, Australia, and Brazil.^[16–18]^ Additionally, no meta-analysis has been conducted on the transition from MHO to MAO status based on available evidence. There is still no convincing evidence regarding the effects of interventions on the metabolic profile for MHO subjects as well. Therefore, the current analysis aimed to firstly collect and estimate the prevalence of MHO individuals in obesity by race/ethnicity, then quantitatively review the risk of progress from MHO phenotype to unhealthy state. At last, we assessed the effects of energy-restricted diet intervention, with or without exercise co-intervention, for the MHO subjects.

## 2 Materials and methods

### 2.1 Search strategy and selection criteria

This meta-analysis was carried out in accordance with PRISMA (preferred reporting items for systematic reviews and meta-analyses) guidelines.^[19]^ Ethical approval was not sought for this study because all data came from the published studies, and no individual-level data were used. A systematic literature search was performed using the database of Medline, EMBASE, Web of Science, and Cochrane library, the screened articles limited to English-language articles published between inception on December 31, 2016. The following truncated search terms were used with various combinations: *obesity, metabolic health, metabolically healthy, metabolic syndrome, prevalence, prevalence, metabolic abnormality, cardiometabolic, and intervention.*

All potentially eligible studies were considered for further review, which was scanned by one of the authors and then confirmed by another author. The two authors also retrieved and assessed potentially relevant publications, and the reference lists of the screened literatures as well as previous relevant reviews and meta-analyses were also checked to identify additional publications of interest.

The full-text of potentially eligible articles was obtained to review eligibility for inclusion. The following criteria were used to select articles for inclusion in the review. For the meta-analysis of the prevalence of MHO in all the obesity: (1) the study reported the prevalence for MHO in the obese population; (2) the participants were adults (age ≥ 18 years); (3) the study was populational-based cross-sectional or longitudinal study; (4) the study used the body mass index (BMI) to define obesity; (5) the definition of metabolic health was based on the cutoffs of the general metabolic components defined by the Third Report of National Cholesterol Education Program's Adult Treatment Panel (NCEP ATP III) or International Diabetes Federation (IDF), including systolic blood pressure (SBP), diastolic blood pressure (DBP), high-density lipoprotein cholesterol (HDL-C), triglycerides (TG), and fasting plasma glucose (FPG).

For the meta-analysis depicted the natural course of MHO: (1) the study was prospective, and the study population was adults (age ≥ 20) at baseline; (2) participants were metabolically healthy obesity, alone or combined with metabolically healthy normal-weight subjects; (3) the study used BMI to define obesity; (4) the outcome was developing one or more metabolic abnormalities defined by the MetS criteria of ATP III or IDF, including high SBP or DBP, low HDL-C, high TG, and high FPG.

For the meta-analysis of intervention studies of MHO: (1) the study was in intervention design, and participants were adults (age ≥ 18); (2) the intervention strategy included energy-restricted diet, alone or combined with exercise; (3) the participants were in MHO phenotype, alone or combined with MAO subjects; (4) the study provided the pre- and postintervention metabolic parameters, including SBP or DBP, HDL-C, TG, and FPG.

### 2.2 Data extraction

Data from each study were extracted by one author, cross-checked by another and imputed into a code sheet. Information extraction from each article included the following items: publication data (first author's name, year of publication), country of the participants, the age of participants, definition of metabolic health, obese criteria, the number of MHO and the obese subjects, the length of follow-up duration, the number of participants who had an event, the change of metabolic parameters in the intervention studies. If there was disagreement, the third investigator resolved it.

### 2.3 Data analysis

We estimated the prevalence of MHO in obesity with 95% confidence intervals (CIs) overall and by regions. The rates were first transformed into arcsine square root. The transformed data were fitted for a random effects model. The articles reporting multiple time points concerning to the incidence of MA for MHO individuals, only the final time point was used in the analyses. The extracted incidence rates of MA from each individual study were pooled by conducting random effects meta-analysis. The reported or calculated relative risks (RRs) from each individual study were pooled to determine the risk of developing MA for MHO compared with the MHNW subjects. Concerning to the meta-analysis of intervention studies relating to MHO individuals, the pre- and postintervention values (mean and standard deviation [SD]), as well as mean differences and associated SDs, were extracted. The anthropometric outcomes of interest were weight, and metabolic parameters included SBP, DBP, HDL-C, TG, and FPG. The mean differences and standardized mean differences (SMDs) were both estimated for each metabolic parameter within each study and then pooled using a random-effects model. The SMD was used to determine the magnitude of the effect, where <0.2 was defined as trivial, 0.2–0.4 as small, 0.4–0.8 as moderate, and >0.8 as large.^[20]^

The *I*^2^ statistic was used to describe the percentage of between-study heterogeneity. *I*^2^ described the percentage of total variation across studies that was due to heterogeneity rather than sampling error and ranges between 0% (no inconsistency) and 100% (high heterogeneity) with values of 25%, 50%, and 75% suggesting low, moderate, and high heterogeneity.^[21]^ A random-effects model was used when heterogeneity was moderate or high. Publication bias was evaluated by the Egger tests. Sensitivity analyses were carried out by excluding one trial at a time to test the robustness of the pooled results. STATA 12 (Stata Corp, College Station, TX) were used for all the analyses, and two-sided *P* < .05 was considered statistically significant.

## 3 Results

### 3.1 Literature search and article selection

**Figure 1:**
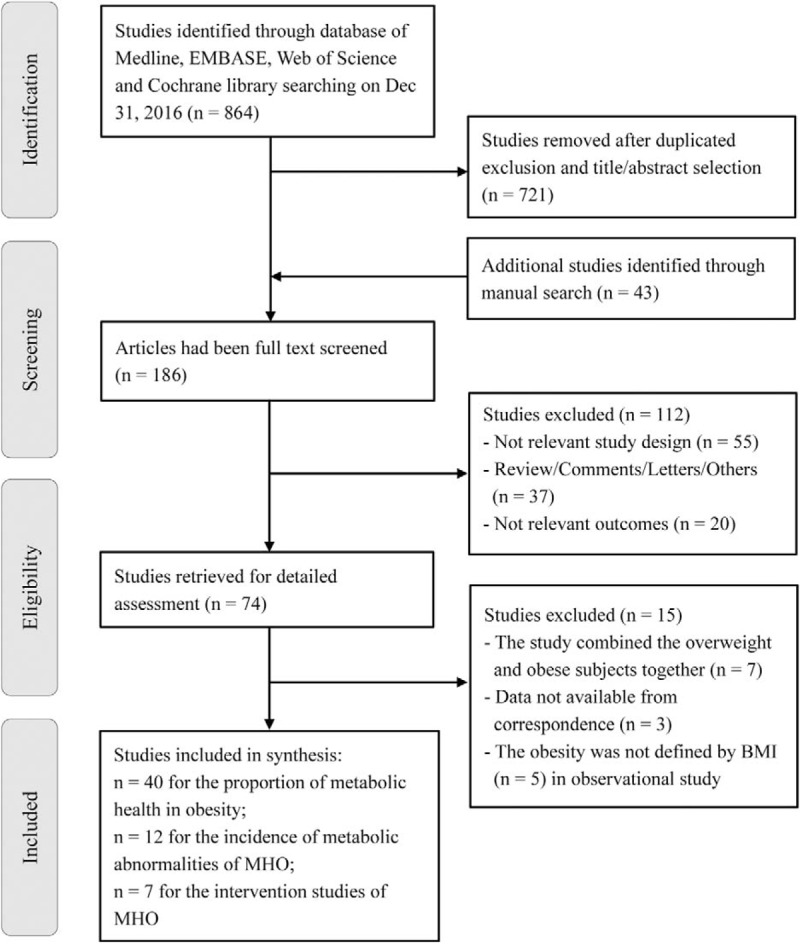
Outline of the study selection process for the meta-analysis.

Our initial search identified 864 potentially relevant articles, as shown in the flow chart reported in Figure 1. After screening of the title or abstract and exclusion of duplicates, 186 articles remained for further evaluation. We excluded 112 studies and remained 74 studies for the following detailed assessments. Seven studies were excluded for they combined the overweight and obese subjects together. For three studies, we corresponded with authors, but data was not available.^[22–24]^ Five studies were excluded, because they did not define obesity by using the criteria of BMI. Finally, 40 population-based provided the prevalence of MHO in obesity,^[13–18,25–58]^ 12 cohort studies^[5,6,33,59–67]^ had reported the incidences of MA in MHO subjects, and seven studies^[8,10,68–72]^ evaluated the effects of lifestyle intervention for MHO.

### 3.2 The prevalence of MHO individuals in obesity

**Figure 2:**
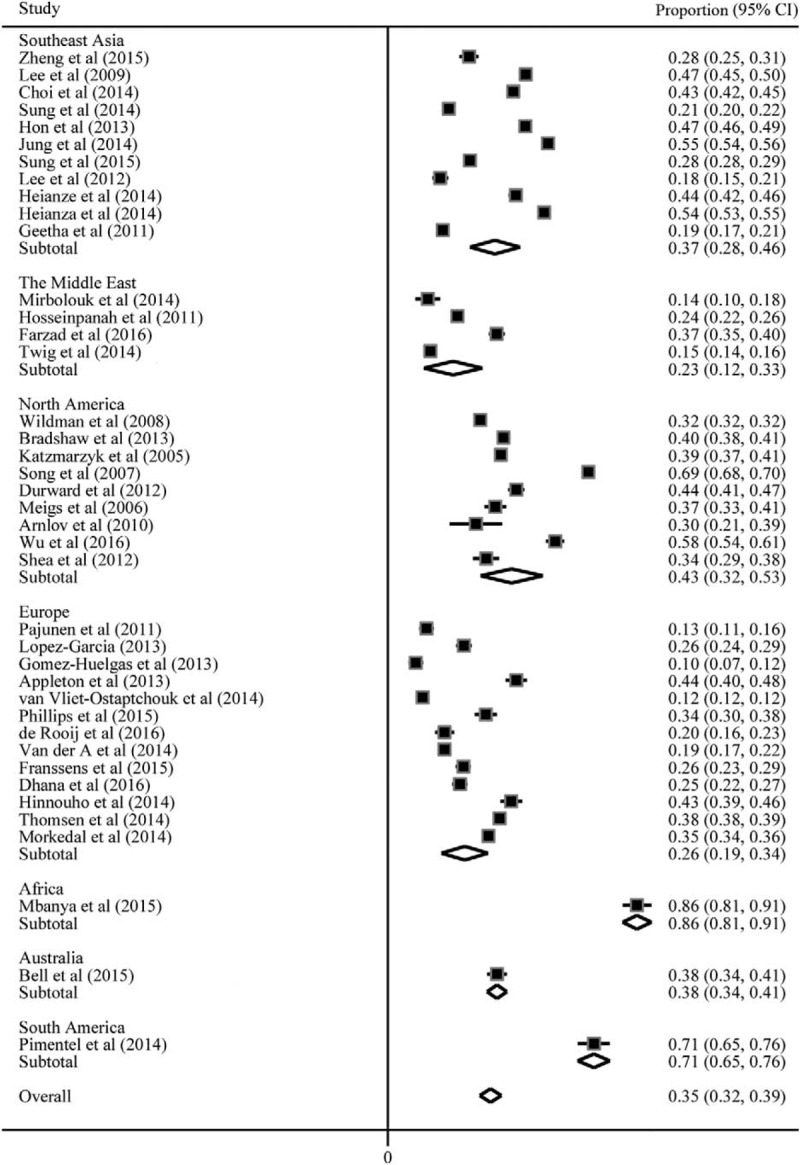
Meta-analyses of the prevalence of metabolically healthy obesity in obesity.

Study characteristics are provided in Supplementary Table 1. The individual studies were carried out in the Southeast Asia (n = 11), the Middle East (n = 4), North America (n = 9), Europe (n = 13), Africa (n = 1), Australia (n = 1), and South America (n = 1). The overall prevalence of MHO ranged between 0.13 and 0.86. The meta-analysis of the total prevalence of MHO in obesity was 0.35 (95%CI: 0.32, 0.39) with a high level of heterogeneity (*I*^2^ = 99.0%) (Fig. 2). The prevalence of MHO was higher in women (0.38, 95%CI: 0.35, 0.42) than that in men (0.31, 95%CI: 0.28, 0.34). The prevalence of MHO was higher in younger age (0.38, 95%CI: 0.32, 0.39) than that in elderly (0.32, 95%CI: 0.19, 0.45) (see Supplementary Table 2). There were dramatic differences in the prevalence among different areas. The highest prevalence of MHO in obesity were in Africa (0.86, 95%CI: 0.81, 0.91) and South America (0.71, 95%CI: 0.65, 0.76). The prevalence of MHO was similar among Southeast Asia (0.37, 95%CI: 0.28, 0.46), North America (0.43, 95%CI: 0.32, 0.53), and Australia (0.38, 95%CI: 0.34, 0.41). The lowest prevalence of MHO was in Europe (0.26, 95%CI: 0.19, 0.34) and the Middle East (0.23, 95%CI: 0.12, 0.33) (Fig. 2).

For sensitivity analysis, each single study was removed at a time and the analysis as repeated on the remaining studies to assess whether our findings were affected by the excluded studies. All the results appeared to be robust to the influence of individual study (see Supplementary Figure 1). No significant publication bias was detected by the Egger tests for all analyses, all *P* values for a two-sided test were > .05 (see Supplementary Table 3).

### 3.3 The risk of developing MA for MHO individuals

**Figure 3:**
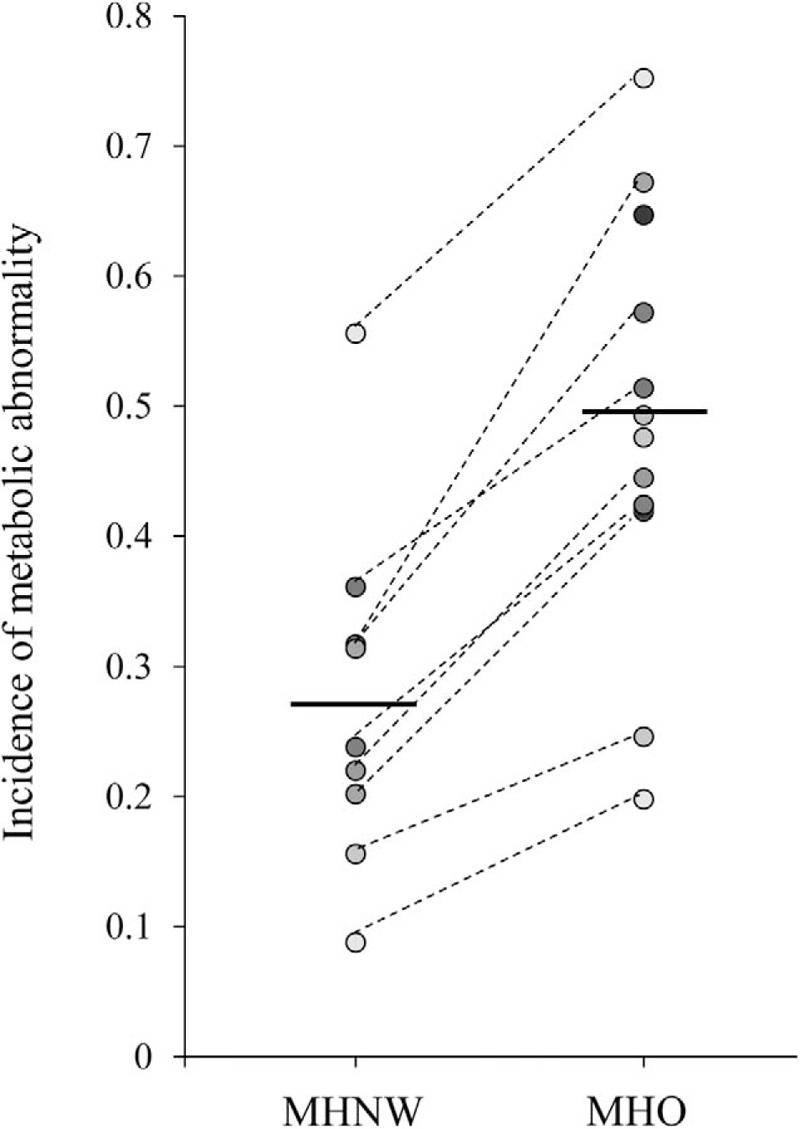
The incidence of one or more metabolic abnormalities in metabolically healthy obesity and metabolically healthy normal-weight subjects in each study. MHNW = metabolically healthy normal-weight; MHO = metabolically healthy obesity.

Study characteristics are provided in Supplementary Table 4. The individual studies were carried out in the Europe (n = 5), Asia (n = 5), and United States (n = 2). Totally, the meta-analysis included 5914 MHO and 26203 MHNW participants. The mean age of the participants ranged from 36 to 63. The follow-up duration ranged from 3 to 10 years. Pooled incidences of MA were 0.49 (95%CI: 0.38, 0.60) and 0.27 (95%CI: 0.18, 0.36) for MHO and MHNW individuals, respectively (see Fig. 3, Supplementary Figures 2 and 3). The pooled incidence of MA for MHO individuals was not varied so much in Asian (0.47, 95%CI: 0.27, 0.67) and Caucasian (0.49, 95%CI: 0.43, 0.56).

**Figure 4:**
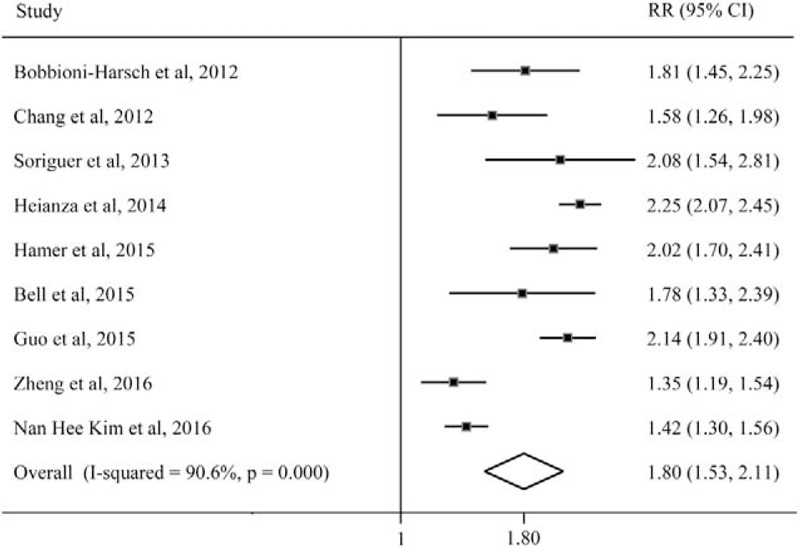
The risk of incident one or more metabolic abnormalities for metabolically healthy obesity compared with metabolic healthy normal-weight subjects. CI = confidence interval; RR = relative risk.

The MHO subjects had significantly higher risk of incident MA compared with the MHNW subjects (pooled RR = 1.80, 95%CI: 1.53, 2.11; *I*^2^ = 90.6%) (Fig. 4). The meta-regression and subgroup analysis showed that the heterogeneity might not attribute to different follow-up duration, ethnic group, and the criteria of metabolic health (Table 1). However, the pooled RR was robust and consistent in the studies with longer follow-up duration (pooled RR = 2.07, 95%CI: 1.89, 2.26; *I*^2^ = 0.0%) and the studies with the subjects in Caucasian (pooled RR = 2.03, 95%CI: 1.88, 2.20; *I*^2^ = 0.0%).

**Table 1:**
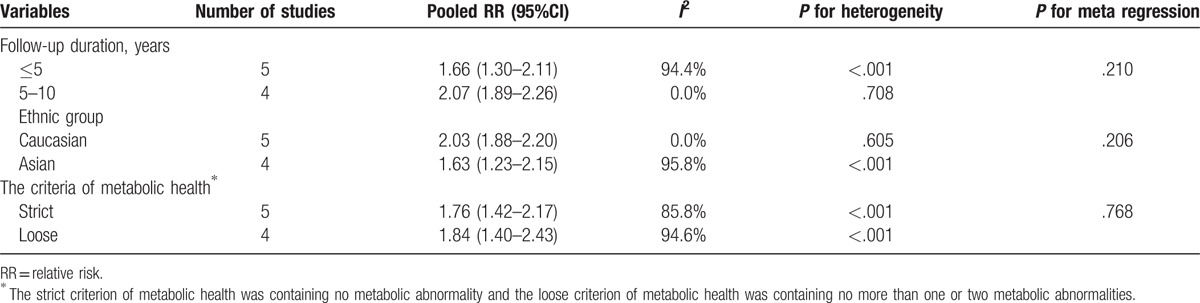
The pooled relative risk of incident metabolic abnormalities for metabolically healthy obesity stratified by variables.

For sensitivity analysis showed that all the results appeared to be robust to the influence of individual study (see Supplementary Figures 4 and 5). No significant publication bias was detected by the Egger tests for all analyses, all *P* values for a two-sided test were > 0.05 (see Supplementary Table 3).

### 3.4 The effects of intervention on the metabolic components of MHO individuals

Study characteristics are provided in Supplementary Table 5. The studies were carried out in the Europe (n = 3), Asia (n = 1), and North America (n = 3). The meta-analysis included 357 MHO and 360 MAO participants. The mean age of the participants ranged from 37 to 56. The duration of interventions varied in length from 2 to 9 months. The intervention strategies include energy-restricted diet intervention, alone (n = 3) or combined with exercise intervention (n = 4).

Following intervention, there was a most certain moderate reduction on weight for both MHO and MAO individuals (Table 2). However, there was only small significant reduction in DBP for MHO individuals (mean difference = −2.41 mm Hg, 95%CI: −4.04, −0.78; SMD = −0.34, 95%CI: −0.57, −0.11). No significant improvement in the other metabolic components was observed for MHO participants. Unlike the effects of intervention for MHO individuals, the effects of intervention on the metabolic components were certain and significant for MAO subjects, except the change of HDL-C.

**Table 2:**
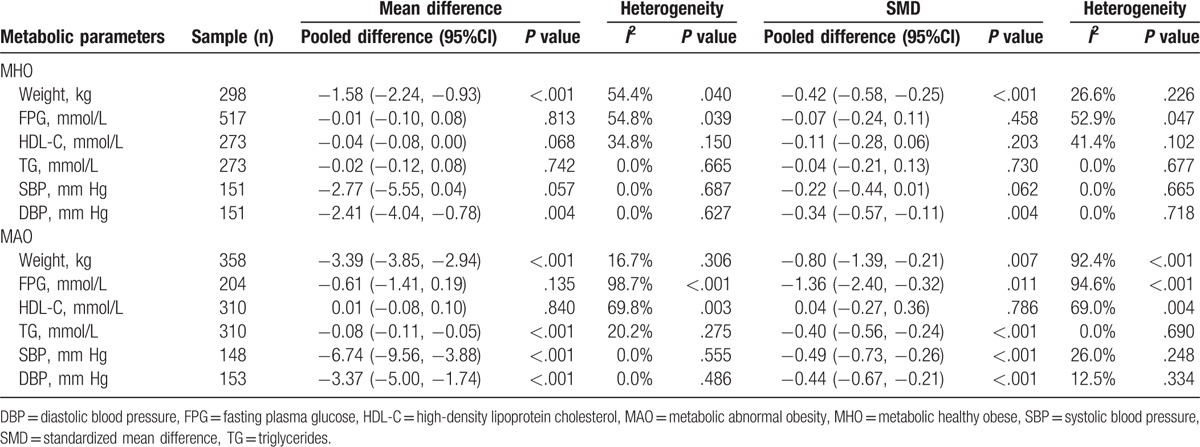
The effects of intervention on metabolic healthy obese and metabolic abnormal obese individuals.

Then, we compared the changes of the weight and metabolic components between MHO and MAO. The results showed that there were significant differences in weight change (mean difference = 1.81, 95%CI: 1.04, 2.58) (*P* < .001), FPG change (mean difference = 0.60, 95%CI: 0.08, 1.12) (*P* = .02), and marginal significant difference in SBP change (mean difference = 3.95, 95%CI: −0.06, 7.96) (*P* = .05) between MHO and MAO subjects. No significant publication bias was detected by the Egger tests for all analyses (see Supplementary Table 3).

## 4 Discussion

The current epidemic of worldwide obesity was one of the greatest public health issues of this century. Given the current approaches to prevent obesity have limited success; this begs the question of whether stratifying obese individuals into MHO and MAO subgroups may offer new opportunities in obesity treatment. Our meta-analysis, based on estimates from 40 studies, showed that one-third of the obese population was in metabolic health, with a large variation in different areas. However, MHO individuals had higher risk of progressing to the metabolic abnormal state than the MHNW counterparts, and half of the MHO individuals would lose their metabolic health over time. Additionally, we summarized the effects of traditional lifestyle intervention on the metabolic profile for the MHO and MAO subjects. Even though the pooled analysis of the intervention studies seemed that the intervention did not played significant role on metabolic parameters for MHO individuals as it did for MAO; however, it still suggested to be effective for its role of counterbalance the adverse effect of obesity.

Rey-López et al had once summarized the prevalence of MHO in worldwide in 2014. They compared the MHO prevalence in different race/ethnicity. Agree with them, we also found that MHO prevalence was higher in population from Asia than the subjects in Europe, but a little lower than that in North America. This finding was paradoxical because Asians are especially prone to visceral fat accumulation and are at higher risk of developing diabetes than other races.^[73]^ Wang et al^[12]^ also conducted meta-analysis concerning to the prevalence of MHO. Differ from our study, they summarized it as the prevalence of MHO in general population. However, we considered that it had more implications to report it as the percentages of metabolic health subjects in obesity, since the obese prevalence was varied in different populations. There were only two studies reported the prevalence of MHO in South America (Brazil) and Africa (Cameroon), respectively. Both of them reported much higher prevalence of MHO in obesity than the people in other areas. They attributed it to the distinct patterns of fat distribution by ethnicity, the obese people in Brazilian and Cameroon might have less visceral adipose tissue and less ectopic fat deposition than the obese people in other ethnicity.^[16,18]^ The study-specific difference of prevalence might attribute to age, ethnicity, sample size, or environmental factors and genetics, and inconsistent definition of metabolic health. In the present meta-analysis, we selected the studies which used similar metabolic syndrome definition of NCEP ATP III or IDF to define metabolic health. However, there were still large variations in MHO prevalence. It is important to note that, among these studies, the obesity was defined by BMI, which did not discriminate between fat and lean body mass; thus, individuals of short stature or muscular build might be misclassified. Despite study design and population differences, the observed variation in MHO prevalence reported both in the comparative studies and meta-analyses highlights that a very large number of obese individuals were temporary in metabolically healthy state.

Even though previous reports on the risks of CVD and mortality in MHO individuals were contradictory, three meta-analysis had proven that a modest increase in CVD risk for MHO subjects.^[3,74,75]^ By following 3.5 million individuals, Caleyachetty et al^[76]^ confirmed the CVD risk for MHO subjects with unprecedented statistical power. The associations between elevated risk of diabetes and MHO phenotype was certain.^[4]^ It was in accordance with the present study that the MHO patients had a significantly higher risk of developing one or more metabolic abnormalities (RR: 1.80) compared with the MHNW individuals. Therefore, it contributed to the higher risk of developing other major diseases. Agree with our speculation, the study of Appleton et al. had shown that the consistent MHO state was associated with a nonsignificant increased risk of diabetes compared with the MHNW phenotype, and the increased risk of diabetes for the MHO individuals was attributed to those who progressed from the MHO to MAO over time.^[49]^ However, Heianza et al^[33]^ observed that both the maintaining MHO and progressing to MAO were associated with higher risk of incident diabetes. The same conclusion also obtained by Kim et al.^[67]^ In their longitude study, the risk for diabetes and CVD was still elevated in subjects with persistent MHO phenotype.

Despite accumulating evidence suggesting that MHO was transient condition, little attention had been given regarding the variables that predict deterioration from MHO to MAO phenotype. Because the characterization of the factors that distinguish those who progress to or maintain MHO from those who transition from MHO to MAO might uncover potential intervention targets. Some studies suggested that MHO subjects were more likely to be younger, female, and of non-Hispanic white ethnicity than subjects in MAO.^[2]^ Our previous cross-sectional analyses had been published that compared with MAO subjects, MHO had consumed more fruits and vegetables, engaged in more intensive physical activity.^[57]^ Hwang et al^[66]^ also compared daily energy intake and physical activity between subjects who remained in MHO and subjects with converted to MAO. They found that energy intake and expenditure were not major determinants for conversion to a metabolically unhealthy phenotype in subjects with MHO, because of no differences in daily energy intake physical activity between the two groups. However, the traditionally energy-restricted diet and exercise intervention had been proven to be effective in improving metabolic profile for obesity. In our study, we also observed that the intervention played significant role for MAO patients, but the effects was not apparent in MHO individuals despite moderate weight loss in them. Two studies supported the theory that MHO and MAO individuals should require a different treatment approach, because they reacted differently.^[9,68]^ The study of Shin et al^[68]^ showed significant reductions in blood lipid profiles for both MHO and MAO after intervention, but the levels of CRP and oxidized LDL fell only in MAO individuals, not in MHO. However, it was also possibly that the MHO individuals were already “metabolically” healthy at baseline despite having an important excess body weight, therefore the metabolic parameters did not change a lot. Different from the traditional lifestyle intervention, gastric bypass was the most potent medical therapy to cure obesity. One study had observed that the gastric bypass surgery had a strong positive metabolic effect in both MHO and MAO subjects.^[77]^ However, the gastric bypass surgery was always suitable for morbid obese subjects currently. In the study of Stefan et al^[78]^ suggested that MHO subjects would benefit from the thiazolidinediones treatment, which might resistant to obesity-induced atherovascular complications. Further studies are needed to monitor the long-term clinical implications and effective intervention strategies for MHO individuals, so as to precisely preventing the development of metabolic abnormalities for cost-efficacy reasons.

The limitations of this study should be considered. First, it should be noted that we relied on variously reported energy-restriction interventions, with or without exercise. Even though the lifestyle interventions were generally benefitted for obese subjects, the intensity of intervention was different from study to study. Furthermore, the durations of intervention were ranged from 2 to 9 months among these original intervention studies, some studies might not last long enough for detecting a biological effect reflected by changes in biomarker concentrations. However, the effects of intervention on MAO patients were evidently and consistent. Therefore, we speculated the factors associated with intervention strategies might not weaken our conclusion. Finally, our analysis was restricted by the data provided within the available studies each having its own methodological characteristics and potential drawbacks. In this respect, we should acknowledge the differences in the assay quality measurements and range of investigated biomarkers.

In conclusion, this systematic review and meta-analysis of population-based investigation and intervention studies suggests that almost one-third of the obese subjects are in metabolic health state. However, MHO phenotype has higher risk of developing metabolic abnormalities, and half of them will convert to metabolic unhealthy status. Therefore, it is still needed to suggest them to maintain or adopt a healthy lifestyle to counterbalance the effects of obesity and keep them in a metabolically healthy condition.

## Supplementary Material

Supplemental Digital Content
