# Supplementary material for: The prevalence, metabolic risk and effects of lifestyle intervention for metabolically healthy obesity: a systematic review and meta-analysis: A PRISMA-compliant article
Source: Medicine (Baltimore). 2017 Nov 27;96(47):e8838. doi: 10.1097/MD.0000000000008838 (PMC5708991; doi:10.1097/MD.0000000000008838)
Supplement: Supplemental Digital Content [file medi-96-e8838-s001.doc]

Supplementary Figure 1. Sensitivity analysis of the meta-analysis of the prevalence of metabolically healthy obesity.


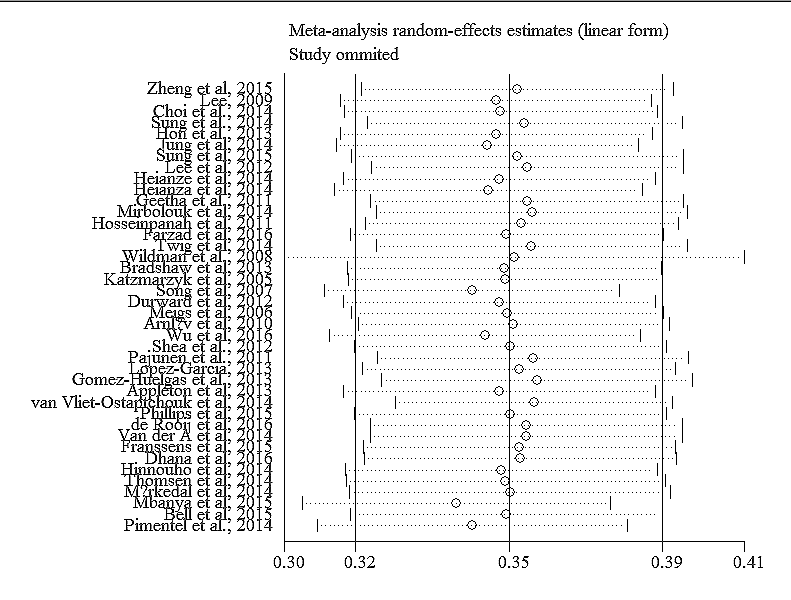


Supplementary Figure 2. Meta-analyses of the incidence of metabolic abnormalities of metabolic healthy obesity subjects.


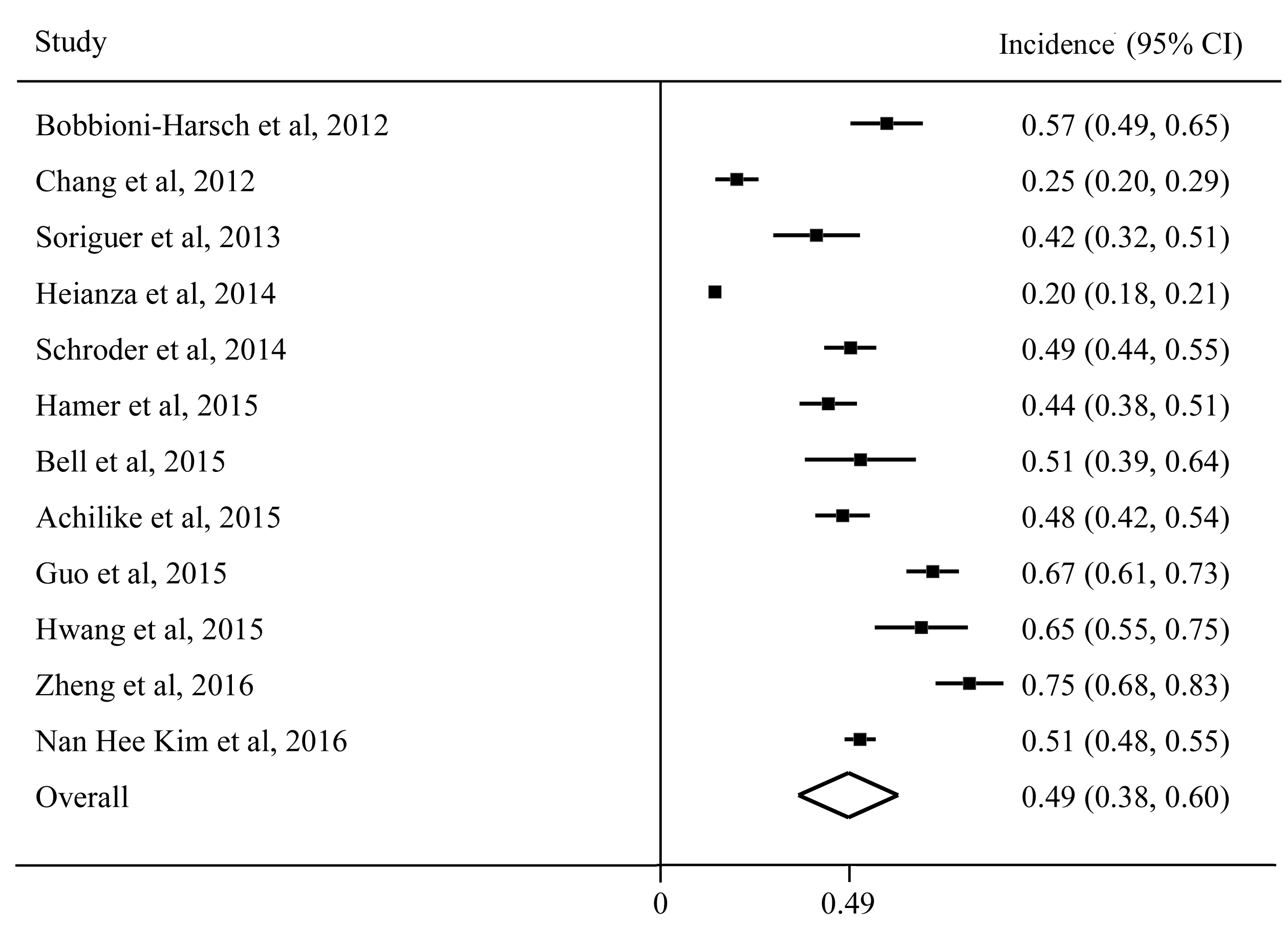


Supplementary Figure 3. Meta-analyses of the incidence of metabolic abnormalities of metabolic healthy normal-weight subjects.


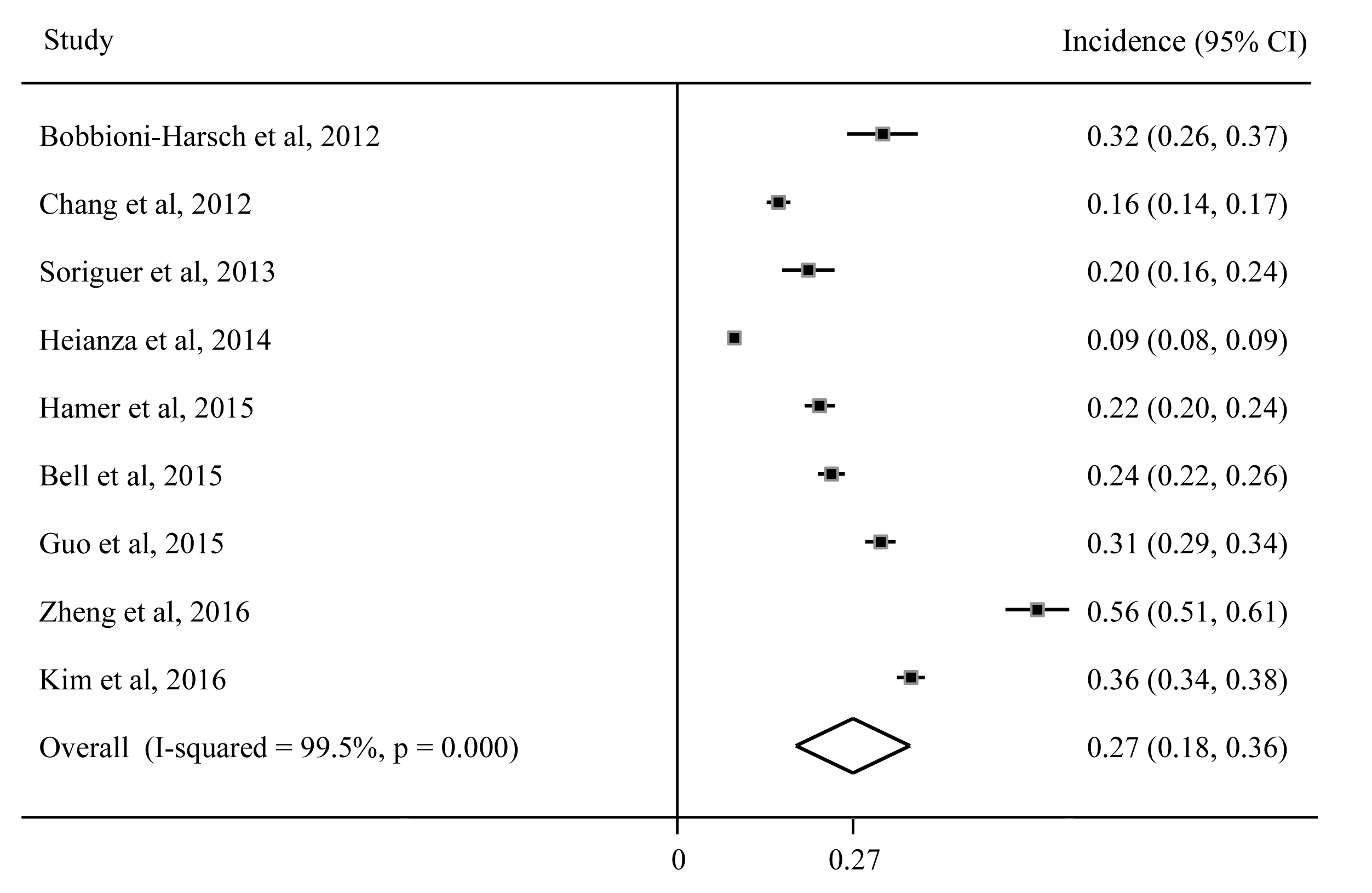


Supplementary Figure 4. Sensitivity analysis of the meta-analysis of incidence metabolic abnormalities for MHO subjects.


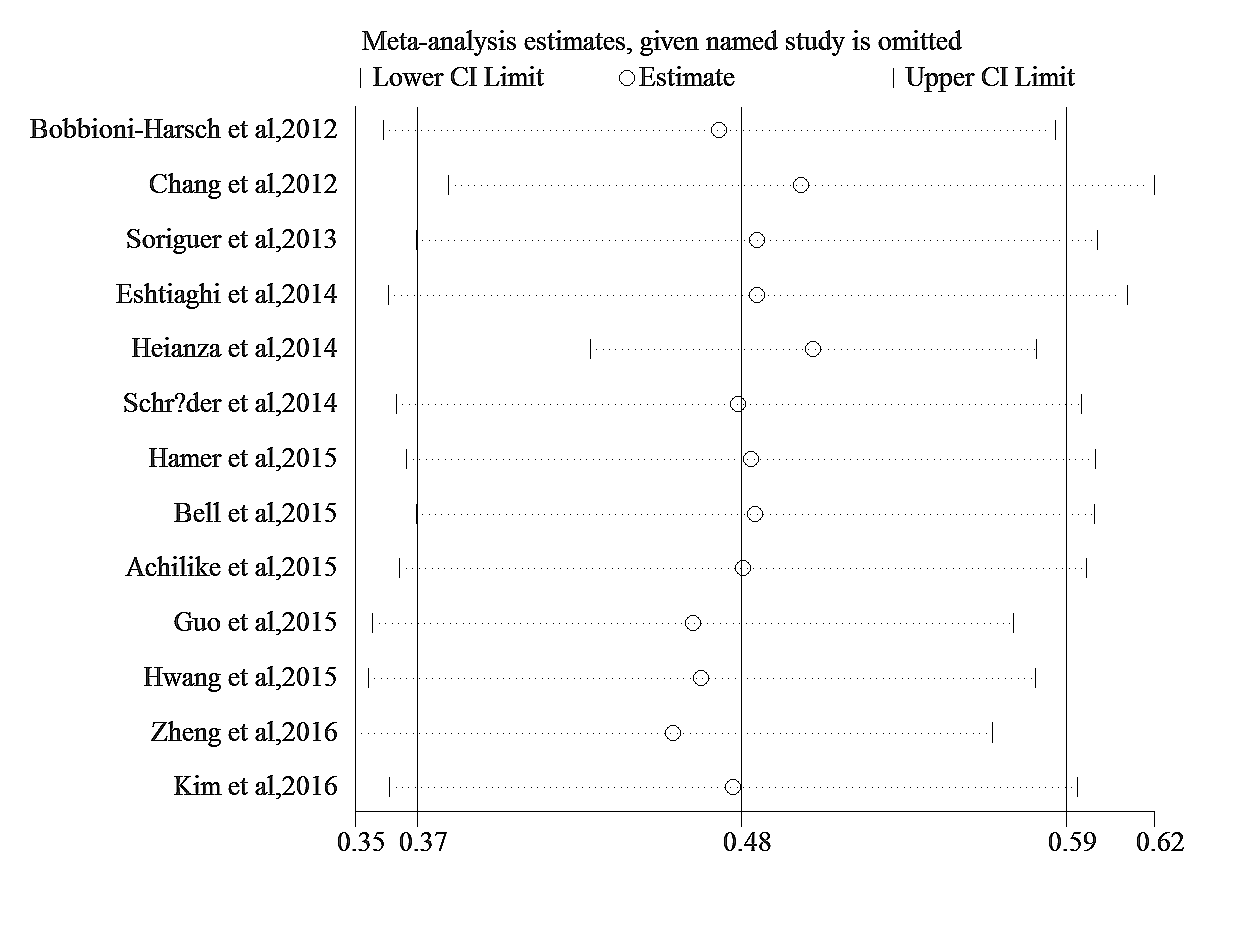


Supplementary Figure 5. Sensitivity analysis of the meta-analysis of incidence metabolic abnormalities for metabolic healthy normal-weight subjects.


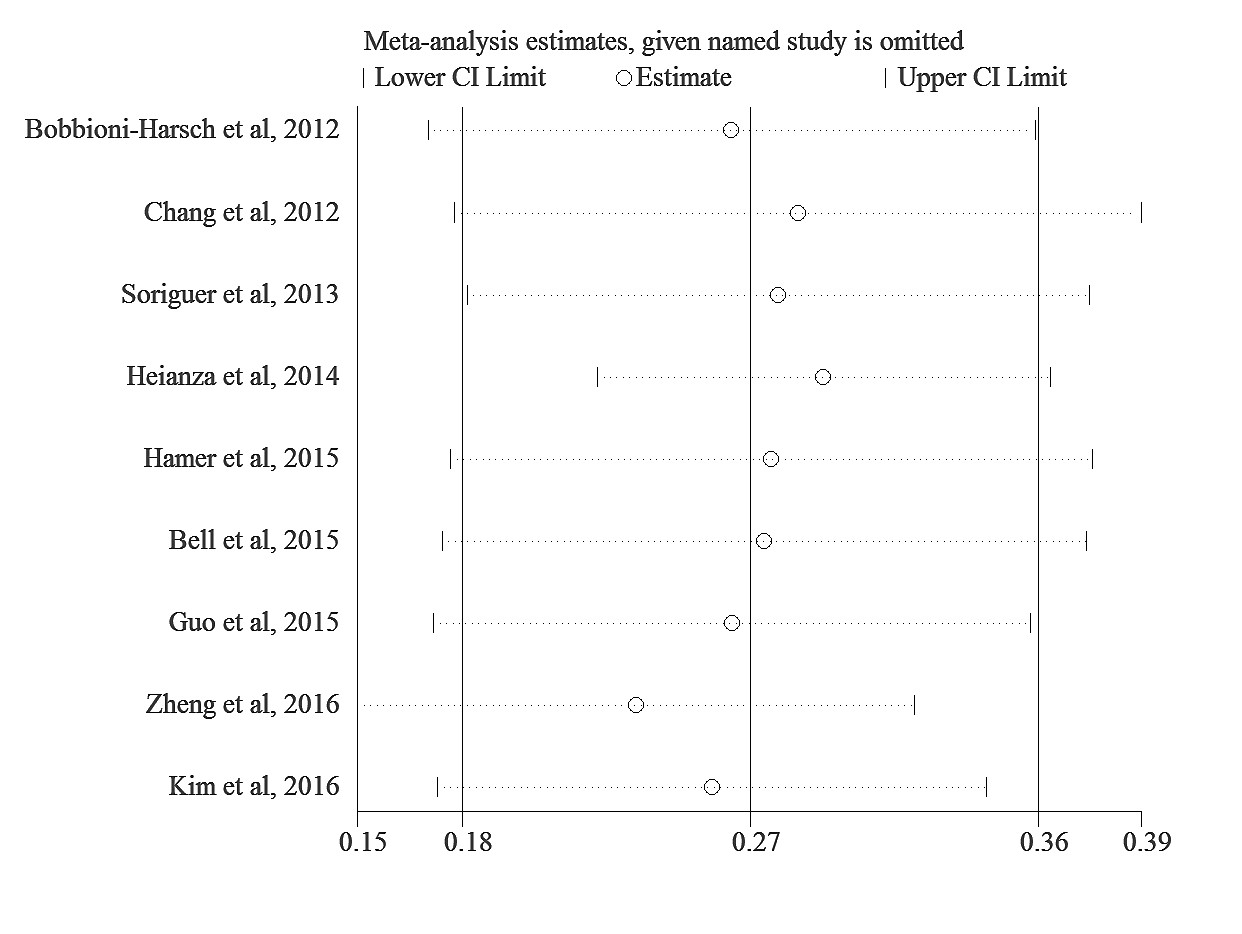


Supplementary table 1. The characteristics of included studies concerning to the prevalence of metabolic health individuals in obesity

| Studies | Country | Participants` age | Obese definition | Metabolically healthy definition | Number of MHO | Number of Obesity | Response rate |
| --- | --- | --- | --- | --- | --- | --- | --- |
| *Southeast Asia* | |  |  |  |  |  |  |
| Zheng et al, 2015 | China | Aged 35 to 72 | BMI≥ 28 kg/m^2^ | ≤ 1 components of MetS by ATP III criteria | 196 | 702 | NA |
| Lee, 2009 | South Korea | Aged over 20 | BMI≥ 25 kg/m^2^ | Not diagnozed as MetS by ATP III criteria | 799 | 1685 | NA |
| Choi et al, 2014 | South Korea | Aged over 60 | BMI≥ 25 kg/m^2^ | Not diagnozed as MetS by ATP III criteria | 1630 | 3770 | NA |
| Sung et al, 2014 | South Korea | Mean age 42 | BMI≥ 25 kg/m^2^ | Absence of any components of the ATP-III | 945 | 4488 | NA |
| Hon et al, 2013 | South Korea | Mean age 42.3 | BMI≥ 25 kg/m^2^ | ≤ 1 components of MetS by ATP III criteria | 2412 | 5096 | NA |
| Jung et al, 2014 | South Korea | Mean age 47.5 | BMI ≥ 25 kg/m^2^ | ≤ 1 components of MetS by ATP III criteria | 6039 | 10940 | NA |
| Sung et al, 2015 | South Korea | Mean age 40.2 | BMI ≥ 25 kg/m^2^ | Absence of any MetS risk factors | 5730 | 20247 | NA |
| Lee et al, 2012 | South Korea | Mean age 48.7 | BMI ≥ 25 kg/m^2^ | Absence of any components of the ATP-III criteria | 135 | 746 | NA |
| Heianze et al, 2014 | Japan | Mean age 48 | BMI ≥ 25 kg/m^2^ | ≤ 1 components of MetS by IDF criteria | 719 | 1634 | NA |
| Heianza et al, 2014 | Japan | Mean age 50 | BMI ≥ 30 kg/m^2^ | ≤ 1 components of MetS by IDF criteria | 3053 | 5675 | 99.0% |
| Geetha et al, 2011 | India | Age over 20 | BMI≥ 25 kg/m^2^ | Not diagnozed as MetS by ATP III criteria | 312 | 1647 | 90.4% |
| *North America* | |  |  |  |  |  |  |
| Wildman et al, 2008 | United States | Age over 20 | BMI≥ 30 kg/m^2^ | ≤ 1 components of MetS by ATP III criteria | 19500000 | 61400000 | NA |
| Bradshaw et al, 2013 | United States | Age 45 to 64 | BMI≥ 30 kg/m^2^ | Not diagnozed as MetS by ATP III criteria | 1602 | 4024 | 93% |
| Katzmarzyk et al, 2005 | United State | Mean age 43 | BMI ≥ 30 kg/m^2^ | ≤ 2 components of MetS by ATP III criteria | 1019 | 2619 | NA |
| Song et al, 2007 | United State | Age over 45 | BMI ≥ 30 kg/m^2^ | ≤ 2 components of MetS by ATP III criteria | 2925 | 4239 | NA |
| Durward et al, 2012 | United State | Mean age 37 | BMI ≥ 30 kg/m^2^ | ≤ 2 components of MetS by ATP III criteria | 513 | 1161 | NA |
| Meigs et al, 2006 | United State | Mean age 53 | BMI ≥ 30 kg/m^2^ | ≤ 2 components of MetS by ATP III criteria | 236 | 638 | NA |
| Arnlöv et al, 2010 | United State | Mean age 50 | BMI ≥ 30 kg/m^2^ | ≤ 2 components of MetS by ATP III criteria | 30 | 99 | 82.0% |
| Wu et al, 2016 | Mexica | Mean age 46 | BMI ≥ 25 kg/m^2^ | ≤ 1 components of MetS by ATP III criteria | 544 | 944 | NA |
| Shea et al, 2012 | Canada | Age over 21 | BMI≥ 30 kg/m^2^ | Not diagnozed as MetS by ATP III criteria | 142 | 420 | NA |
| *The Middle East* | | |  |  |  |  |  |
| Mirbolouk et al, 2014 | Iran | Mean age 70 | BMI ≥ 30 kg/m^2^ | ≤ 2 components of MetS by Joint Interim Statement (2009) criteria | 35 | 254 | NA |
| Hosseinpanah et al, 2011 | Iran | Mean age 47 | BMI ≥ 30 kg/m^2^ | ≤ 2 components of MetS by Joint Interim Statement (2009) criteria | 408 | 1700 | NA |
| Farzad et al, 2016 | Iran | Age over 20 | BMI ≥ 25 kg/m^2^ | ≤ 2 components of MetS criteria of Modified Joint Interim Statement (2009) | 452 | 1206 | NA |
| Twig et al, 2014 | Israel | Mean age 30.9 | BMI ≥ 30 kg/m^2^ | Absence of any components of the ATP-III | 631 | 4293 | NA |
| *Europe* |  |  |  |  |  |  |  |
| Pajunen et al, 2011 | Finland | Age 45 to 74 | BMI≥ 30 kg/m^2^ | Not diagnozed as MetS by IDF criteria | 94 | 703 | 64.0% |
| Lopez-Garcia, 2013 | Spain | Mean age 46.9 | BMI≥ 30 kg/m^2^ | ≤ 1 components of MetS by IDF criteria | 283 | 1082 | 78.7% |
| Gomez-Huelgas et al, 2013 | Spain | Age 18 to 80 | BMI≥ 30 kg/m^2^ | Not diagnozed as MetS by IDF criteria | 50 | 520 | NA |
| Appleton et al, 2013 | Europe | Mean age 42 | BMI ≥ 30 kg/m^2^ | ≤ 1 components of MetS by IDF criteria | 297 | 672 | 69.4% |
| van Vliet-Ostaptchouk et al, 2014 | Europe | Mean age 52.6 | BMI≥ 30 kg/m^2^ | ≤ 1 components of MetS by IDF criteria | 3387 | 28077 | NA |
| Phillips et al, 2015 | Irish | Mean age 60 | BMI≥ 30 kg/m^2^ | Not diagnozed as MetS by ATP III criteria | 196 | 581 | 67.0% |
| de Rooij et al, 2016 | Netherlands | Age 40 to 75 | BMI≥ 30 kg/m^2^ | Not diagnozed as MetS by ATP III criteria | 107 | 547 | NA |
| Van der A et al, 2014 | Netherlands | Age 20 to 59 | BMI ≥ 30 kg/m^2^ | Absence of any components of the ATP-III | 264 | 1354 | 45.0% |
| Franssens et al, 2015 | Netherlands | Mean age 56 | BMI ≥ 30 kg/m^2^ | ≤ 2 components of MetS criteria of ATP III and high hs-CRP | 293 | 1127 | 58.9% |
| Dhana et al, 2016 | Netherlands | Mean age 68 | BMI ≥ 30 kg/m^2^ | ≤ 2 components of MetS criteria of Joint Interim Statement (2009) | 260 | 1048 | NA |
| Hinnouho et al, 2014 | United Kingdom | Age 35 to 55 | BMI ≥ 30 kg/m^2^ | ≤ 1 components of MetS by ATP III criteria | 279 | 656 | 73.0% |
| Thomsen et al, 2014 | Denmark | Age 20 to 100 | BMI ≥ 30 kg/m^2^ | ≤ 2 components of MetS by ATP III criteria | 4416 | 11500 | 45.0% |
| Mørkedal et al, 2014 | Norway | Adults | BMI ≥ 30 kg/m^2^ | ≤ 2 components of MetS by IDF criteria | 3479 | 10055 | 69.0% |
| *Africa* |  |  |  |  |  |  |  |
| Mbanya et al, 2015 | Cameroon | Age 24 to 74 | BMI≥ 30 kg/m^2^ | ≤ 1 components of MetS by ATP III criteria | 163 | 190 | 93.0% |
| *Australia* | |  |  |  |  |  |  |
| Bell et al, 2015 | Australia | Age over 45 | BMI≥ 30 kg/m^2^ | Not diagnozed as MetS by ATP III criteria | 290 | 772 | 84.8% |
| *South America* |  |  |  |  |  |  |  |
| Pimentel et al, 2014 | Brazilian | Mean age 43 | BMI≥ 30 kg/m^2^ | ≤ 1 components of MetS by ATP III criteria | 183 | 258 | NA |

ATP III, the Third Report of National Cholesterol Education Program's Adult Treatment Panel; IDF, International Diabetes Federation; MetS, metabolic syndrome; BMI, body mass index; NA, not available

Supplementary table 2. The prevalence of MHO stratified by age and gender

| Variables | Number of studies | Prevalence of MHO | 95%CI | *I*^2^ | *P* value for heterogeneous |
| --- | --- | --- | --- | --- | --- |
| Gender |  |  |  |  |  |
| Men | 33 | 0.31 | 0.28, 0.34 | 99.6% | < 0.001 |
| Women | 32 | 0.38 | 0.35, 0.42 | 99.6% | < 0.001 |
| Age |  |  |  |  |  |
| Mean age below 50 | 14 | 0.38 | 0.32, 0.39 | 99.7% | < 0.001 |
| Mean age over 50 | 14 | 0.32 | 0.19, 0.45 | 99.8% | < 0.001 |
| General age (>18) | 12 | 0.35 | 0.31, 0.39 | 99.3% | < 0.001 |

MHO, metabolically healthy obesity

Supplementary table 3. Publication bias assessment for the pooled results by Egger's test

|  | Coefficient | Standard error | *P* value |
| --- | --- | --- | --- |
| The prevalence of MHO in obesity | 1.371 | 3.839 | 0.723 |
| The risk of incident MA for MHO | -0.915 | 4.967 | 0.859 |
| The effect of intervention on weight for MHO | -2.495 | 1.251 | 0.103 |
| The effect of intervention on weight for MAO | -3.426 | 5.564 | 0.571 |
| The effect of intervention on glucose for MHO | 1.318 | 1.682 | 0.469 |
| The effect of intervention on glucose for MAO | 1.286 | 7.204 | 0.867 |
| The effect of intervention on HDL-C for MHO | 1.581 | 1.485 | 0.328 |
| The effect of intervention on HDL-C for MAO | 0.469 | 2.063 | 0.829 |
| The effect of intervention on TG for MHO | -0.712 | 0.924 | 0.470 |
| The effect of intervention on TG for MAO | -0.338 | 0.917 | 0.727 |
| The effect of intervention on SBP for MHO | -1.784 | 1.151 | 0.219 |
| The effect of intervention on SBP for MAO | 1.585 | 1.516 | 0.373 |
| The effect of intervention on DBP for MHO | -1.934 | 0.895 | 0.119 |
| The effect of intervention on DBP for MAO | 1.560 | 1.394 | 0.345 |

MA, metabolically abnormalities; MHO, metabolically healthy obesity; MAO, metabolically abnormal obesity; SBP, systolic blood pressure; DBP, diastolic blood pressure; TG, triglycerides; HDL-C, high density lipoprotein cholesterol

Supplementary table 4. The characteristics of included studies concerning to the natural course of metabolic heathy individuals

| Author, year | Country | Participants` age | Follow-up duration | The definition of obesity | The definition of metabolic health | Sample size of MHO | Sample size of MHNW |
| --- | --- | --- | --- | --- | --- | --- | --- |
| Bobbioni-Harsch et al, 2012 | 14 European countries | Mean age 44 | 3 years | BMI ≥ 25 kg/m^2^ | Absence of any MetS risk factors of ATP III | 152 | 284 |
| Chang et al, 2012 | Korea | Mean age 36 | 5 years | BMI ≥ 25 kg/m^2^ | Absence of any MetS risk factors of ATP III, plus HOMA-IR < 2.5; hsCRP<1.0 mg; absence of fatty liver | 329 | 1496 |
| Soriguer et al, 2013 | Spain | Mean age 40 | 6 years | BMI ≥ 30 kg/m^2^ | Absence of any MetS risk factors of ATP III and HOMA-IR < 90th percentile | 105 | 377 |
| Heianza et al, 2014 | Japan | Mean age 48 | 2 years | BMI ≥ 25 kg/m^2^ | < 2 of MetS risk factors of IDF | 3131 | 17334 |
| Schröder et al, 2014 | Spain | Mean age 45 | 10 years | BMI ≥ 25 kg/m^2^ | Absence of diabetes, hypertension, hypercholesterolemia, hypertriglyceridemia, and low HDL-C and high LDL-C | 301 | - |
| Hamer et al, 2015 | United Kindom | Mean age 63 | 8 years | BMI ≥ 30 kg/m^2^ | < 2 of MetS risk factors of Wildman RP | 243 | 1206 |
| Bell et al, 2015 | United Kindom | Age 39 - 62 | 10 years | BMI ≥ 30 kg/m^2^ | < 2 of MetS risk factors of ATP III including HOMA-IR < 90th percentile | 66 | 1677 |
| Achilike et al, 2015 | United States | Age 25 - 64 | 8 years | BMI ≥ 30 kg/m^2^ | < 2 of MetS risk factors of ATP III including HOMA-IR < 5.13 | 275 | - |
| Guo et al, 2015 | United States | Mean age 54 | 10 years | BMI ≥ 30 kg/m^2^ | Absence of any MetS risk factors of ATP III | 260 | 1499 |
| Hwang et al, 2015 | Japan | Mean age 50 | 10 years | BMI ≥ 25 kg/m^2^ | < 3 of MetS risk factors of ATP III | 85 | - |
| Zheng et al, 2016 | China | Mean age 52 | 5 years | BMI ≥ 24 kg/m^2^ | Absence of any MetS risk factors of ATP III | 133 | 392 |
| Kim et al, 2016 | Korea | Mean age 51 | 5 years | BMI ≥ 25 kg/m^2^ | < 2 of MetS risk factors of ATP III | 834 | 1938 |

ATP III, the Third Report of National Cholesterol Education Program's Adult Treatment Panel; IDF, International Diabetes Federation. MA, metabolic abnormalities; MHO, metabolic healthy obese; MHNW, metabolic healthy normal-weight; HOMA-IR, homeostasis model assessment-estimated insulin resistance

Supplementary table 5. The characteristics of included intervention studies of metabolic heathy obese and metabolic abnormal obese individuals

| Author, year | Country | Participants age | Participants | The definition of overweight/obesity | The definition of metabolic health | Intervention duration | Intervention |
| --- | --- | --- | --- | --- | --- | --- | --- |
| Shin et al, 2006 | Korea | Mean age 39 | 23 MHO and 106 MAO women | BMI ≥ 25 kg/m^2^ | < 2 of MetS risk factors of ATP III and the study of Brochu et al | 3 months | Energy-restricted diet intervention |
| Janiszewski et al, 2010 | Canada | Mean age 56 | 66 MHO and 43 MAO | Waist circumference ≥ 88 cm in women and 102 cm in men | < 2 of MetS risk factors of ATP III | 3 months | Energy-restricted diet or exercise intervention |
| Kantartzis et al, 2011 | Germany | Mean age 47 | 26 MHO and 77 obese insulin-resistant individuals | BMI ≥ 30 kg/m^2^ | Insulin sensitivity estimated from the OGTT | 9 months | Energy-restricted diet and exercise intervention |
| Ruiz et al, 2012 | Spain | Mean age 37 | 53 MAO and 25 MHO women premenopausal | Waist circumference ≥ 88 cm in women | < 2 of MetS risk factors of ATP III including HOMA-IR ≤ 90th percentile | 3 months | Energy-restricted diet intervention |
| Dalzill et al, 2014 | Canada | Mean age 53 | 55 MHO and 79 MAO | Fat mass percentage ≥ 25% in men and ≥ 35% in women | < 2 of MetS risk factors of ATP III | 9 months | High-intensity interval training and mediterranean diet intervention |
| Gardner et al, 2015 | United States | Mean age 42 | 31 insulin resistence and 30 insulin sensitive obesity | BMI ≥ 28 kg/m2 | Area under the curve of insulin concentrations based on oral glucose tolerance test | 6 months | Low-fat or low-carbohydrate diets intervention |
| Rondanelli et al, 2015 | Italy | Mean age 42 | 103 MHO subjects | BMI ≥ 30 kg/m2 | Healthy lipid profile and HOMA-IR ≤ 1.95 | 2 months | Energy-restricted diet and exercise intervention |

ATP III, the Third Report of National Cholesterol Education Program's Adult Treatment Panel; IDF, International Diabetes Federation. HOMA-IR, homeostasis model assessment-estimated insulin resistance; OGTT, oral glucose tolerance test.
